# Novel pathogenic variants in *KIT* gene in three Chinese piebaldism patients

**DOI:** 10.3389/fmed.2022.1040747

**Published:** 2022-11-10

**Authors:** Chen Wang, Yingzi Zhang, Xuyun Hu, Lijuan Wang, Zhe Xu, Huan Xing

**Affiliations:** ^1^Department of Dermatology, National Center for Children’s Health, Beijing Children’s Hospital, Capital Medical University, Beijing, China; ^2^Department of Dermatology, Shunyi Maternal and Children’s Hospital of Beijing Children’s Hospital, Beijing, China; ^3^Beijing Key Laboratory for Genetics of Birth Defects, MOE Key Laboratory of Major Diseases in Children, Genetics and Birth Defects Control Center, National Center for Children’s Health, Beijing Children’s Hospital, Beijing Pediatric Research Institute, Capital Medical University, Beijing, China

**Keywords:** piebaldism, *KIT* gene, whole-exome sequencing, café-au-lait macule, genotype-phenotype correlation

## Abstract

**Background:**

Piebaldism is a rare autosomal dominant disease, and roughly 75% patients had *KIT* gene mutations. Up to date, approximately 90 *KIT* mutations causing piebaldism were reported.

**Methods:**

To identify *KIT* gene mutations in three pediatric piebaldism patients from different families and explore the genotype-phenotype correlation, peripheral blood DNA were collected from probands and their parents. Whole-exome sequencing was performed to detect potential disease-causing variants in the three probands. Putative variants were validated by Sanger sequencing.

**Results:**

Heterozygous variants of c.2469_2484del (p.Tyr823*), c.1994G > C (p.Pro665Leu), and c.1982_1983insCAT (p.662_663insIle) in *KIT* gene were detected in three probands. These variants were all novel and classified as pathogenic/likely pathogenic variants according to the interpretation guidelines of American College of Medical Genetics and Genomics and the Association for Molecular Pathology. The probands carrying variants located in tyrosine kinase domain exhibited a more severe phenotype.

**Conclusion:**

The piebaldism in three families was caused by novel heterozygous *KIT* variants. The severity of phenotypes is related with the types and locations of different mutations. Our results further provided evidence for genetic counseling for the three families.

## Introduction

Piebaldism (OMIM 172800) is a rare autosomal dominant disorder of congenital depigmentation characterized by patches of white skin and hair in a distinct ventral midline pattern (1). The white patches are involved in the areas of middle frontal, chest, abdomen, and limbs. The color and range remain stable throughout life. Some patients also have café-au-lait macules (CALMs) and intertriginous freckling, as well as extra-cutaneous manifestations such as, epitheliomas, occasional deafness and rare Hirschsprung disease. The incidence is unknown, yet it is estimated to be less than 1 in 20,000 (2). Both males and females are affected equally.

Piebaldism is caused by mutations in *KIT* or *SNAI2*. These two genes are involved in the development, survival, and migration of melanocyte precursors (3). Roughly 75% of piebaldism patients are caused by mutations in the *KIT* gene (OMIM 164920) located on chromosome 4q12 (4), while other patients may have mutations in alternative genes like the *SNAI2* gene (OMIM 172800) located on chromosome 8q11 (5). *KIT* encodes a transmembrane tyrosine kinase receptor KIT for stem cell factor, which is important in the melanogenesis pathway (6). The receptor KIT belongs to type III transmembrane receptor tyrosine kinase family. It is composed of an amino-terminal extracellular ligand-binding domain (EC), a single transmembrane domain (TB), and an intracellular tyrosine kinase (TK) domain. Mutations in *KIT* gene lead to abnormal melanocyte migration and the absence of melanocytes.

In order to identify underlying genetic etiology of piebaldism patients and further extend the phenotype and mutation spectra, we performed next-generation sequencing for three Chinese piebaldism families. In this study, we uncovered three novel pathogenic/likely pathogenic *KIT* variants (c.2469_2484del, c.1994G > C, and c.1982_1983insCAT). Our study provided the basis for genetic counseling of three piebaldism families. The results further elucidated the genotype-phenotype correlation that mutations in TK domain caused severe clinical manifestations.

## Materials and methods

### Patient recruitment

Three individuals clinically suspected as piebaldism were recruited from Beijing Children’s Hospital. The age of these patients ranged from 5 months to 3 years. All the probands presented with varying degrees of skin pigmentation and poliosis. Written informed consents were obtained from the minors’ legal guardian for the publication of any potentially identifiable images or data included in this article. This study was approved by the Institutional Medical Ethics Committee of Beijing Children’s Hospital, Capital Medical University [(2022)-E-196-R] and conducted according to the Declaration of Helsinki.

### Whole-exome sequencing

Peripheral blood of the probands and their parents were collected, and genomic DNA was extracted by Blood Genomic DNA Kit (TransGen, Beijing). Whole-exome sequencing (WES) was performed for three patients (mean depth > 100×). The library was sequenced on NovaSeq (Illumina, San Diego, America) and aligned to the GRCh38/hg38 human reference sequence using Burrows-Wheeler Aligner (BWA) with the MEM algorithm. BAM files were generated by Picard. Sequence reads were recalibrated by Realigner Target Creator in Genome Analysis Toolkit (GATK), and sequence variants were called by GATK Haplotype Caller. Copy Number Variants (CNVs) were called by read-depth strategy by CNVkit. Variants were annotated and filtered by software of Flash Analysis (fa.shanyint.com). Variants were classified following the American College of Medical Genetics and Genomics and the Association for Molecular Pathology (ACMG/AMP) interpretation standards and guidelines (7). Putative pathogenic variants detected by next-generation sequencing (NGS) were confirmed by Sanger sequencing. According to the WES results, Sanger sequencing was used to verify the gene mutation sites of the probands and their parents. Primer premier 5 software is used to design primers.

### Histopathological examination

Skin biopsy was performed on a 1.0 cm × 0.5 cm fusiform skin tissue from the depigmented lesion on the right upper arm of Proband 1. The skin tissue was placed in normal saline and then fixed in 4% paraformaldehyde overnight. The fixed tissue was washed, dehydrated, and finally embedded with paraffin. Sections of 5-μm thickness were cut by a microtome (RIWARD, Shenzhen) and stained with hematoxylin and eosin (H&E). Skin tissue sections were observed under the light microscope (Keyence, China).

## Results

### Clinical manifestations

**FIGURE 1:**
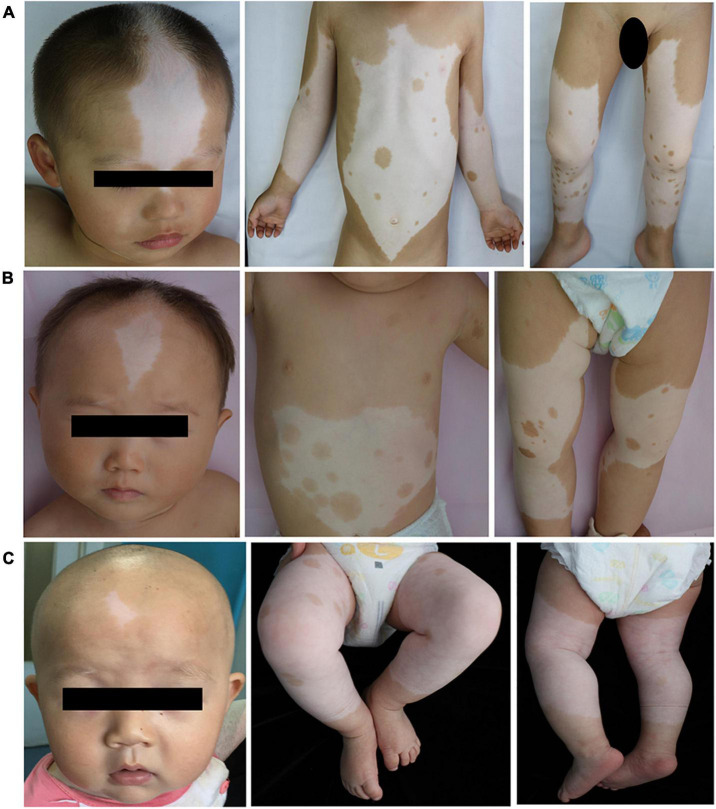
Main clinical manifestations of the three patients. Depigmented patches were involved in middle frontal, chest, abdomen, limbs of the patient 1 **(A)**, 2 **(B)** and 3 **(C)**. Normal pigment islands were scattered on trunk, and extremities. Café-au-lait macules were noticed on the right lower limb of Patient 1 and the front chest, left armpit, right lower limb of Patient 2.

Clinical features of these three patients with piebaldism were summarized in Table 1. No other findings such as facial deformity, heterochromia iridis, deafness, or anemia were noticed. Patient 1 was a 2-year and 8-month-old boy with congenital leukodermal patches of the forehead, ventral abdomen, limbs, and a white forelock. The size of the patches increased proportionally with age. His father had a similar phenotype (Figure 1A). Patient 2 was a 9-month-old girl. She presented with poliosis and skin depigmentation patches on the forehead, trunk, limbs since birth. Both her parents had normal phenotype (Figure 1B). Patient 3, a 5-month-old boy, had a white forelock and unpigmented skin patches on the forehead, trunk, bilateral arms, and legs since birth. His father had similar physical symptom, and other family members were not affected (Figure 1C).

**TABLE 1:** Clinical manifestations of probands 1–3.

Proband	Age	Gender	Mutation	Origin	Involved area						
					
					Forehead	Forelock	Front chest	Abdomen	Back	Upper limbs	Lower limbs
1	2 years and 8 months	Male	c.2469_2484del(p.Tyr823*)	Paternal	+	+	+	+	–	+	+
2	9 months	Female	c.1994C > T(p.Pro665Leu)	*De Novo*	+	–	–	+	+	+	+
3	5 months	Male	c.1982_1983insCAT (p.662_663insIle)	Paternal	+	+	+	–	–	+	+

### Molecular genetic analysis

**FIGURE 2:**
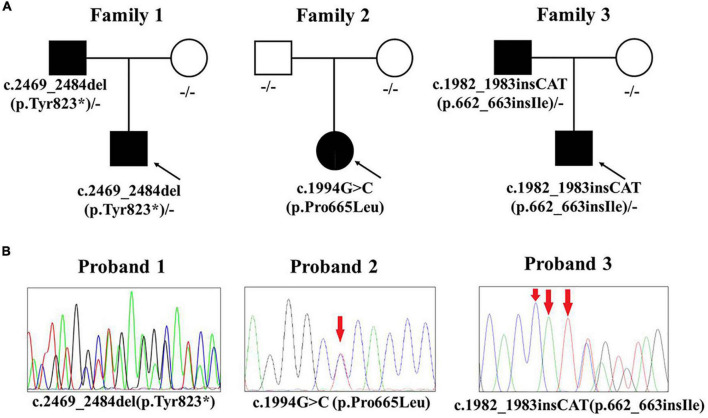
*KIT* mutations of the three piebaldism patients. **(A)** Pathogenic variants and their origin in the three families. **(B)** Sanger sequencing results of three pathogenic variants.

The results of WES showed that heterozygous variants of *KIT* gene (NM_000222.2) were detected in all three patients (Table 1 and Figure 2). Patient 1 had a paternal variant c.2469_2484del (p.Tyr823*) in exon 17, resulting in a termination codon at position 823. It is expected to lead to nonsense-mediated mRNA decay and lost the function. Patient 2 had a *de novo* variant c.1994C > T (p.Pro665Leu). Pro665 located on catalytic domain of tyrosine-protein kinase and was highly conserved according to predicting tools such as phyloP, GERP + + and REVEL. All SIFT, Polyphen-2, CADD and LRT had Damaging/Deleterious prediction of p.Pro665Leu. At the same site, p. Pro665Ser was already reported in another Chinese piebaldism family (8). Patient 3 had a paternal variant c.1982_1983insCAT (p.662_663insIle) which was also located on catalytic domain of tyrosine-protein kinase. All the three variants were absent in population databases including gnomAD, Exome Sequencing Project (ESP) and 1000G, and not reported in pervious literature. According to the ACMG/AMP interpretation standards and guidelines, c.2469_2484del (PVS1 + PM2 + PP4) and c.1994C > T (PS2 + PM1 + PM2 + PM5 + PP3 + PP4) were classified as pathogenic variants, c.1982_1983insCAT was classified as likely pathogenic variant (PM2 + PM4 + PP3 + PP4).

### Pathological examination results

**FIGURE 3:**
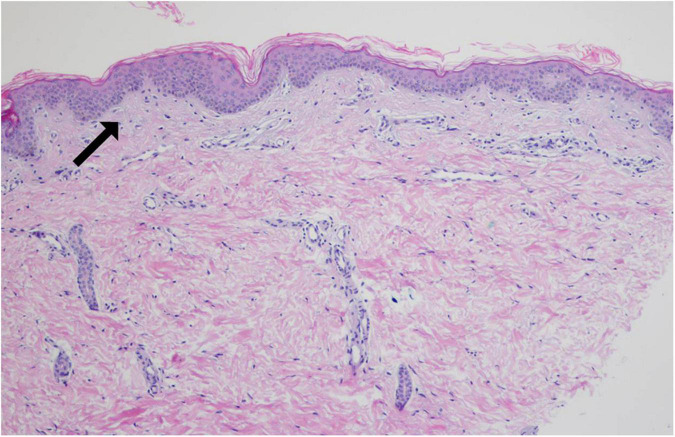
Pathological biopsy result of Patient 1. The arrow indicates that there is no melanocyte in stratum basal of the depigmented area (hematoxylin-eosin staining, ×200).

The skin biopsy specimen taken from Proband 1 revealed: atrophy of epidermis, vanishment of melanocytes and melanin pigment among basal cells, lymphocytes infiltration in the perivascular regions and elastic fiber degeneration (Figure 3). It is consistent with the pathological manifestations of piebaldism.

## Discussion

In this study, we report three Chinese cases with pathogenic/likely pathogenic variants in *KIT*. All these variants were novel variants. These data expand the mutation spectrum of the *KIT* gene.

In 1991, Giebel and Spritz first reported that mutations in *KIT* gene could lead to piebaldism (4). To date, approximately 90 mutations in the *KIT* gene were reported in piebaldism according to the database of HGMD (professional 2022.3). For these mutations, 17 were identified in Chinese patients (Supplementary Table 1). The severity of clinical features in piebaldism patients correlate with the type and location of *KIT* gene mutations (9, 10). Dominant-negative inhibition caused by missense mutations in the TK domain could lead to most severe phenotype. The mild piebaldism phenotype is associated with frameshift variants and missense variants occur in the N-terminal EC domain with haploinsufficiency, and some patients do not even develop any clinical manifestations. Truncating mutations located in the intracellular TK domain or any mutations at or near the TB domain result in intermediate severe phenotype, and different patients in a same family may have different phenotypes. The variants of these three probands were all located in TK domain. Compared with patients who carried the variants in EC domain reported in previous literature (11), the clinical manifestations of three probands were more severe, and all of them showed typical white forelock on frontal scalp, relatively large leukoderma on the chest, abdomen, and extremities. The c. 2469_2484del mutation of Proband 1 located in exon 17 caused a termination codon at position 823 in TK domain. The expression product might lead to haploinsufficiency through nonsense-mediated decay (NMD), or dominant-negative by truncating protein. This type of mutation reduced the normal function of KIT by 50–75%, resulting in a more severe phenotype of this patient. The variants of Proband 2 and 3 were closed to ATP-binding sites (E671, C673, and D677) in TK domain. These two variants might decrease ATP-binding ability by changing motif topological structure according to SWISS-MODEL and AlphaFold. The mutated protein partially retained kinase function and caused milder phenotype of Probands 2 and 3 than Proband 1. This milder phenotype was also reported in a Chinese piebaldism patient with *KIT* missense mutation P665S previously (8). However, proving the precise effect of these mutations requires biochemistry, bioinformatics analysis, and *in vitro* experiment. Meanwhile, the influence of modifying genes or environmental factors on penetrance cannot be ruled out, and further studies are needed.

Significantly, in addition to typical dermatology manifestations of piebaldism, two probands (Probands 1 and 2) in this study also had CALMs. CALMs may present at birth or childhood, and are association with several genetic disorders, such as Neurofibromatosis type 1 (NF1), Legius syndrome (12). NF1 is an autosomal dominant disease characterized by CALM, freckling, neurofibroma, and Lisch nodule (13). It is caused by heterozygous mutation in *NF1* gene. Legius syndrome is also an autosomal dominant disorder due to inactivating mutations in *SPRED1* (14). Individuals with Legius syndrome typically have multiple CALMs, intertriginous freckling without neurofibroma or other tumor. In our study, more than six CALMs > 5 mm in size were found on the trunk or limbs of Proband 2, and less than six in Proband 1. No freckling or neurofibroma was found in three probands, and no *NF1* or *SPRED1* mutation was detected by genetic analysis. Among all the affected family members, none of them had CALMs or freckling. Patients with similar skin manifestations have also been reported in the previous literature (15–17). In the reported cases, all piebaldism patients with CALMs had missense *KIT* mutations located in the TK domain. Therefore, some researchers suggested that CALMs might be related to the location and type of *KIT* gene mutation (17–22). In our study, two variants of Probands 1 and 2 were also located in the TK domain. The p.Tyr823* variant identified in Proband 1 was the first truncating variant in piebaldism patient with CALMs. These two variants could lead to the loss of KIT tyrosine kinase function, inadequate phosphorylation of *SPRED1*, and eventually result in the loss of inhibition of Ras/MAPK pathway (23). More cases are needed to determine whether CALM or freckling is an uncommon phenotypic variation in the piebaldism spectrum.

In conclusion, we uncovered genetic etiology of three Chinese piebaldism patients and reported three novel pathogenic/likely pathogenic variants. We found novel variants next to ATP-binding site might cause less severe phenotypes. We also reported the first truncating variant in piebaldism patient causing CALMs. Our results further expanded clinical and variants spectra and provided more evidence to elaborate genotype-phenotype correlation of *KIT* mutation.

## Data availability statement

The datasets presented in this article are not readily available to protect patient privacy and confidentiality. Requests to access the datasets should be directed to the corresponding author/s.

## Ethics statement

This study was approved by the Institutional Medical Ethics Committee of Beijing Children’s Hospital, Capital Medical University [(2022)-E-196-R]. Written informed consent to participate in this study was provided by the participants’ legal guardian/next of kin. Written informed consent were obtained from the minors’ legal guardian for the publication of any potentially identifiable images or data included in this article.

## Author contributions

HX and ZX designed the research and supervised the study. CW collected cases and followed up patients. YZ analyzed the data and wrote the manuscript. XH revised the manuscript. LW collected cases. All authors contributed to the article and approved the final version.
